# Supplementary material for: Multimodal interference-based imaging of nanoscale structure and macromolecular motion uncovers UV induced cellular paroxysm
Source: Nat Commun. 2019 Apr 10;10:1652. doi: 10.1038/s41467-019-09717-6 (PMC6458150; doi:10.1038/s41467-019-09717-6)
Supplement: Supplementary file 1 — Supplementary Information [file 41467_2019_9717_MOESM1_ESM.pdf]

Supplementary Information

Multimodal interference-based imaging of nanoscale structure and macromolecular motion uncovers UV induced cellular paroxysm

Scott Gladstein et al

Supplementary Note 1: Temporal Interference Theory

A. Autocorrelation of Temporal Interference

Consider a spatially and temporally varying RI object sandwiched between two semi-infinite homogeneous media. The refractive index of the three media are defined as n_0 (representing glass), $n_1(1+n_\Delta(r,t))$ (representing the cell), and n_2 (representing cell culture media). Additionally, we are approixmating that $n_1 = n_2$. When the object is imaged by an epi-illumination bright-field microscope with a small NA (small angle approximation), the time-varying part of intensity reflectance at a single wavelength (normalized by the source) is[1]

$$\delta\mathrm{I}(x',y',k,t) = -2\Gamma\mathcal{I}\{\int_{-\infty}^{+\infty} kn_{1\mathrm{D}}(\mathbf{r},t)e^{-i2kz}dz\} \tag{1}$$

where k is the scalar wavenumber of the illumination light, $\mathbf{r}$ is $(x'/M, y'/M, z)$ inside the sample, M is the objective magnification, $\Gamma = \Gamma_{01}T_{01}T_{10}$ is a Fresnel intensity coefficient, $\mathcal{I}$ denotes the imaginary part of a complex number, $n_{1\mathrm{D}}(\mathbf{r}) = \mathcal{F}_\perp\{T_{k\mathrm{NA}}T_{\mathbf{k_s}}\} \otimes_\perp n_{\Delta t}(\mathbf{r})$, $n_{\Delta t}(\mathbf{r})$ is the time-varying part of n_Δ, $n_{\Delta t} = n_\Delta(\mathbf{r},t) - < n_\Delta(\mathbf{r},t) >_t$, $T_{k\mathrm{NA}}$ is the microscope pupil function–a cone in spatial-frequency space with radius kNA, $T_{\mathbf{k_s}}$ is a windowing function that equals 1 at $\mathbf{k} = \mathbf{k_s}$ and 0 at $\mathbf{k} \neq \mathbf{k_s}$, and $\mathbf{k_s}$ is the scattering wave vector inside the sample.

Since $n_{1\mathrm{D}} = 0$ for negative z ($n_\Delta t$ is zero outside the $(0,L)$ interval in the axial direction), $|\mathcal{I}\{\mathcal{F}_z\{kn_{1\mathrm{D}}\}\}|^2 = |\mathcal{F}_z\{kn_{1\mathrm{D}}\}|^2/2$. Therefore, the temporal autocorrelation (ACF) of δI is expressed as :

$$B_{\delta\mathrm{I}}(x',y',k,\tau) = \int \delta\mathrm{I}(x',y',k,t)\delta\mathrm{I}(x',y',k,t-\tau)dt = 2\Gamma^2k^2L\int e^{-i2kz}B_{n_{1\mathrm{D}}}(\mathbf{r},\tau)dz \tag{2}$$

It follows from the Wiener-Khinchine and convolution theorems, that the autocorrelation of a convolution of two functions equals the convolution of the autocorrelations of those two functions. Therefore,

$$B_{n_{1\mathrm{D}}}(x',y',z,\tau) = \frac{1}{(2\pi)^3}\int B_{n_\Delta t}(x'-x, y'-y, z, \tau)A(x,y)dxdy \tag{3}$$

where $B_{n_\Delta t}$ is the 4D ACF of $n_{\Delta t}$ and A is the ACF of the 2D Airy disk. Note that $B_{\delta\mathrm{I}}(x',y',k,\tau)$ is random since $n_\Delta(\mathbf{r})$ is random. To calculate the sample statistics, we calculate its expected value over (x',y'), denoted as $\widetilde{B_{\delta\mathrm{I}}}(k,\tau)$ Plugging this into equation (2)

$$\widetilde{B_{\delta\mathrm{I}}}(k,\tau) = \frac{2\Gamma^2k^2L}{(2\pi)^4}\int e^{-i\mathbf{kr}}B_{n_\Delta t}(\mathbf{r},\tau)T_{3\mathrm{D}}d^3\mathbf{r}d^3\mathbf{k} \tag{4}$$

where $\mathbf{k}$ is the 3D frequency-space wave vector, $T_{3\mathrm{D}} = T_{k\mathrm{NA}}T_{\mathbf{k_s}}$ a disk with radius kNA in frequency-space centered at $k_z = 2k$. This indicates that the temporal ACF of δI is proportional to the 3D spatial Fourier transform of the 4D spatiotemporal ACF of $n_{\Delta t}$ integrated over $T_{3\mathrm{D}}$.

For the case when $n_{\Delta t}$ has an exponential form of the spatiotemporal ACF,

$$B_{n_\Delta t}(\mathbf{r},\tau) = \sigma^2_{n_\Delta t}e^{-\frac{\mathbf{r}}{l_{\mathrm{cm}}}}e^{-\frac{\tau}{t_{\mathrm{c}}}} \tag{5}$$

where $\sigma_{n_\Delta t}$ is the variance of $n_\Delta t$, l_{cm} is the correlation distance of moving material, and t_{c} is the correlation time. Then,

$$\widetilde{B_{\delta\mathrm{I}}}(k,\tau) = \frac{4\Gamma^2k^4L\mathrm{NA}^2l^3_{\mathrm{cm}}\sigma^2_{n_\Delta t}e^{-\frac{\tau}{t_{\mathrm{c}}}}}{(2\pi)^2(1+4k^2l^2_{\mathrm{cm}})(1+k^2l^2_{\mathrm{cm}}(4+\mathrm{NA}^2))} \tag{6}$$

If the sample is thicker than the depth of focus, then $L = \frac{2\pi n_{\mathrm{i}}}{k\mathrm{NA}^2_{\mathrm{i}}}$. Additionally, the equation can be simplified by assuming $kl_{\mathrm{cm}} << 1$; while this simplification may not be valid at large l_{cm}, it is a useful assumption for understanding and discussing the biological interpretation of the dual-PWS signal.

$$\widetilde{B_{\delta\mathrm{I}}}(k,\tau) = \frac{2}{\pi}\Gamma^2k^3n_{\mathrm{i}}\left(\frac{NA_{\mathrm{c}}}{NA_{\mathrm{i}}}\right)^2l^3_{\mathrm{cm}}\sigma^2_{n_\Delta t}e^{-\frac{\tau}{t_{\mathrm{c}}}} \tag{7}$$

where n_{i} is the refractive index of the immersion oil, $\mathrm{NA_c}$ is the NA of collection and $\mathrm{NA_i}$ is the NA of illumination.

B. Calculating the Fractional Moving Mass

$$\Sigma_{\mathrm{t}}^2 = \widetilde{B_{\delta\mathrm{I}}}(k,0) = \frac{2}{\pi}\Gamma^2 k^3 n_{\mathrm{i}} \left(\frac{\mathrm{NA_c}}{\mathrm{NA_i}}\right)^2 l_{\mathrm{cm}}^3 \sigma_{n_\Delta t}^2 \tag{8}$$

In the approximation of a binary moving material,

$$\sigma_{n_\Delta t}^2 = \Phi(1-\Phi)\left(\frac{n_{\mathrm{m}} - n_1}{n_1}\right)^2 \tag{9}$$

where Φ is the volume fraction of the mobile mass, and n_{m} is the refractive index of moving material. If we assume $\Phi << 1$ and $l_{\mathrm{cm}}^3 = V_{\mathrm{cm}}$, where V_{cm} is the volume of the typical moving macromolecular cluster. Then,

$$\Sigma_{\mathrm{t}}^2 = \frac{2}{\pi}\Gamma^2 k^3 n_{\mathrm{i}} \left(\frac{\mathrm{NA_c}}{\mathrm{NA_i}}\right)^2 \left(\frac{n_{\mathrm{m}} - n_1}{n_1}\right)^2 V_{\mathrm{cm}} \Phi \tag{10}$$

$$\Sigma_{\mathrm{t}}^2 \left(\frac{\pi}{2\Gamma^2 k^3 n_{\mathrm{i}}}\right) \left(\frac{\mathrm{NA_i}}{\mathrm{NA_c}}\right)^2 \left(\frac{n_1}{n_{\mathrm{m}} - n_1}\right)^2 = V_{\mathrm{cm}} \Phi \tag{11}$$

Next, we can normalize Σ_{t}^2 by ρ_0, the density of a typical macromolecular cluster.

$$\Sigma_{\mathrm{t}}^2 \left(\frac{\pi \rho_0}{2\Gamma^2 k^3 n_{\mathrm{i}}}\right) \left(\frac{\mathrm{NA_i}}{\mathrm{NA_c}}\right)^2 \left(\frac{n_1}{n_{\mathrm{m}} - n_1}\right)^2 = \rho_0 V_{\mathrm{cm}} \Phi = m_{\mathrm{c}} \Phi = m_{\mathrm{f}} \tag{12}$$

With this normalization, Σ_{t}^2 is equivalent to m_{f}, referred to as the **fractional moving mass**, which is a measure of mass moving within the sample defined by $m_{\mathrm{c}} = V_{\mathrm{cm}}\rho_0$, the mass of your typical moving macromolecular cluster, and the volume fraction of mobile mass, Φ. To calculate this normalization, we approximate $n_{\mathrm{m}} = 1.43$ as the RI of a nucleosome, $n_1 = 1.37$ as the average RI of a nucleus, and $\rho_0 = 0.55$ g cm^{-3} as the dry density of a nucleosome. It should be noted that Σ_{t}^2 is sensitive to instrument parameters such as depth of field, substrate refractive index, etc. For biological measurements, it is important to use parameters such as m_{f}, where these dependencies are remove through normalization with the proper prefactor calculated above. Additionally, the backscattered intensity is prone to errors along the transverse direction[(2)]. Due to these variations, these parameters are most accurate after calculating the expected value over (x', y').

C. Calculating the Diffusion Coefficient

Under the conditions of a dilute macromolecular solution, the normalized ACF, $\widetilde{B_{\delta\mathrm{I}}}(k,\tau)/\widetilde{B_{\delta\mathrm{I}}}(k,0)$, is equivalent to the *self-intermediate scattering function*, which is the Fourier transform of $G(\mathbf{r},t)$, the Van-Hove self space-time correlation function[(3)], where $G(\mathbf{r},\tau)d^3r$ is the probability that a particle at point $\mathbf{r}$ will suffer displacement within d^3r in time t and is also known as Van-Hove self space-time correlation function.

$$\widetilde{B_{\delta\mathrm{I}}}(k,\tau)/\widetilde{B_{\delta\mathrm{I}}}(k,0) = \mathcal{F}\{G(\mathbf{r},\tau)\} \tag{13}$$

$G(\mathbf{r},t)$ can, to a good approximation, be regarded as a solution to the diffusion equation.

$$\frac{\partial}{\partial t} G(\mathbf{r},\tau) = D\nabla^2 G(\mathbf{r},\tau) \tag{14}$$

where D is the diffusion coefficient. The 3D spatial Fourier transform of equation (14) is

$$\frac{\partial}{\partial t} \frac{\widetilde{B_{\delta\mathrm{I}}}(k,\tau)}{\widetilde{B_{\delta\mathrm{I}}}(k,0)} = -4k^2 D \frac{\widetilde{B_{\delta\mathrm{I}}}(k,\tau)}{\widetilde{B_{\delta\mathrm{I}}}(k,0)} \tag{15}$$

The solution to the differential equation (15) is

$$\frac{\widetilde{B_{\delta\mathrm{I}}}(k,\tau)}{\widetilde{B_{\delta\mathrm{I}}}(k,0)} = e^{-4k^2 D\tau} = e^{-\frac{\tau}{t_{\mathrm{c}}}} \tag{16}$$

Therefore, the diffusion coefficient, $D = \frac{1}{4k^2 t_{\mathrm{c}}}$, can be calculated from the correlation time, t_{c}, of the normalized autocorrelation of the temporal interference.

Supplementary Note 2: Timescale Sensitivity

Theoretically, dual-PWS is sensitive to any moving structure regardless of the speed. Practically, experimental parameters such as exposure time, acquisition time, and sampling rate limit the dynamic processes our instrument is sensitive too. Simulations were performed to explore the effect of these parameters on our measured biomarkers. A random 1-D signal representing our measured interference signal is simulated by taking the inverse Fourier Transform of the square root of the power spectral density from a desired autocorrelation function (ACF) with random noise added in the frequency domain. The variance of the signal (Σ_t^2) was set to one and an exponential decay was used for the ACF with the decay coefficient varied to test different diffusion coefficients. This signal can be simulated at extremely high temporal resolution (< 1 ms) and length (> 20 s) to represent a continuous signal that fully captures all motion. Sampling is independently tested by sampling frames from the high frequency data at various sampling rates. Exposure time can be independently tested by convolving the signal with a rectangular function (or the ACF with a triangular function) with a width defined by 2*exposure time. Exposure time and sampling can be combined by binning the data with a width defined by exposure time; this assumes sampling rate and exposure time are equivalent as they are experimentally. Finally, acquisition time can be tested by cutting off data at the end of our signal, so that the length of the signal is equal to the acquisition time. Unless otherwise specified, the imaging parameters used in this analysis were set to the parameters used in the system validation phantom measurements: 32 ms exposure, 32 ms sampling, and 6.4 s acquisition time.

First, we used this simulation data to explore the effects of these imaging parameters on Σ_t^2. Exposure time decreases Σ_t^2 with a larger drop in magnitude for faster diffusion coefficients (Supplementary Figure 1a). With the imaging parameters and diffusion coefficients used in our

phantom experiments, sampling and acquisition length don't have significant effects. By testing extremely slow diffusion coefficients, we observe that acquisition length has the opposite effect of exposure time, reducing Σ_{t}^2 for slower diffusive processes (Supplementary Figure 1b). By decreasing the sampling rate past our experimental parameters, we observe that sampling doesn't cause a directional change for Σ_{t}^2, but results in increased measurement variability (Supplementary Figure 1c). Because the effect of these parameters is monotonic, this simulation data can be used to correct Σ_{t}^2 values if the diffusion coefficient and imaging parameters are known. These correction factors are used in the system validation to modify the theoretical Σ_{t}^2 values to match the experimental nanosphere phantom measurements. Additionally, this data was used to determine our system theoretical sensitivity range, which was defined as the range of diffusion coefficients where Σ_{t}^2 was >50% of the true Σ_{t}^2; for the nanosphere phantom experiments, this range is $0.065\ \mu M^2 s^{-1} > D > 3.7 * 10^{-5}\ \mu M^2 s^{-1}$.

Experimentally, the instrument sensitivity can be measured by extracting dynamics parameters from a static sample. Measuring m_{f} from static samples provides a lower limit of detectable motion. Note that background subtraction is applied in regular m_{f} calculations, so motion below our sensitivity limit will be close to zero instead of the sensitivity level. Without background subtraction, m_{f} measurements of fixed HeLa cells provide a sensitivity limit of m_{f} = 6.03e-20 ± 1e-21 g (n = 7). Measuring diffusion coefficients from static samples provides an upper limit to our temporal sensitivity assuming the system noise is relatively uncorrelated, resulting in the fastest detectable correlation decay. Similar to theoretical limit of 0.065 $\mu M^2 s^{-1}$ measured through simulations, an experimental limit of D = 0.08 ± 0.001 $\mu M^2 s^{-1}$ (n = 7) was measured using fixed HeLa cells. For this analysis, noise removal, such as background subtraction and removal of pixels with low SNR are not performed as we are trying to measure system noise.

Next, we applied the simulation data to explore the effects of these imaging parameters on measured diffusion coefficients. Unless your function is perfectly exponential and completely noiseless, measuring the diffusion coefficient has the additional complication that results can vary depending on the points/range of the ACF that are used to calculate the decay coefficient. Exposure time causes the first section (equivalent to the width of the exposure time) of the ACF to flatten out resulting in inaccurate decays measured within that section (Supplementary Figure 2ab). Measuring the decay at time lags greater than the exposure time is quite accurate in noiseless data (Supplementary Figure 2c). Unfortunately, when noise is added to the data, it mostly effects the SNR at these larger time lags. While slower diffusive processes can be accurately measured, this noise limits our system from measuring diffusion coefficient for faster diffusive processes (Supplementary Figure 2d). This is consistent with the validation measurements, which accurately matched for slower diffusion coefficients, but diverged for phantoms with faster diffusion coefficients (Supplementary Figure 3).

Supplementary Note 3: High Temporal Resolution System

As a proof of concept that sensitivity to faster processes is only limited by existing hardware, we set up a system capable of capturing temporal interference at speeds up to 1.2ms per frame and ~240 ms per m_f map. This enables sensitivity to much faster cellular motion with a theoretical sensitivity range of $2\,\mu M^2 s^{-1} > D > 0.002\,\mu M^2 s^{-1}$. The UV irradiation experiment was repeated on this system. Mostly consistent with our previous experiments, continuous UV irradiation induced a steady decrease in the fractional moving mass ($\downarrow m_f$) (Supplementary Movie 1, Supplementary Figure 4). Measurements from this system are more sensitive to fast motion and less sensitive to slower motion, changing the actual cellular processes being quantified, suggesting

that this decrease in molecular motion is occurring universally within the cell across a large range of dynamic processes. This data shows that with some slight system modifications the dual-PWS dynamics technique is capable of measuring nanoscale molecular motion with unprecedented temporal resolution.

This data was acquired on a commercial inverted optical microscope (Eclipse Ti-U with perfect focus system, Nikon) with a 120 W broadband LED light source (X-Cite 120LED). The illumination aperture was partially closed, limiting the NA of the illumination light to ~0.55. Scattered light was collected by a 100X 1.49 NA oil immersion objective, then passed through a 532 nm bandpass filter with a 3 nm spectral width (532/3 nm BrightLine Semrock) and sent to a Backside Illuminated Scientific CMOS camera (Prime 95B, Photometrics). Each frame was acquired at a ~1.2 ms acquisition time.

Supplementary Note 4: UV Diffusion Analysis

Accurate calculation of the diffusion coefficient requires sufficient SNR. UV irradiation decrease the motion within cells, as seen in the decrease of m_f, effectively decreasing the SNR needed to accurately calculate D as the experiment proceeds. When analyzed using the procedure described above, the percentage of pixels failing to meet the SNR threshold increased throughout the experiment. This resulted in a different population of pixels being analyzed at each timepoint producing misleading results. To properly calculate changes in D for this experiment, we had to adjust our analysis procedure in the following ways:

1) The SNR threshold was lowered slightly from $\sqrt{2} *$ background signal (as explained above) to $1 *$ background signal to increase the percentage of analyzed pixels.

2) Only pixels that met this threshold for every analyzed measurement in the timeseries were included in the analysis to maintain a consistent pixel population.
3) Any cells with less than 10% of the pixels meeting the above conditions were removed from the dataset.

Using this method there is a tradeoff between the length of the experiment analyzed, the number of pixels per cell that meet the noise threshold, and the number of cells that meet the pixel threshold. To best understand the data, D was calculated for three different experiment lengths: 5.4 minutes (20 UV cells with ~66% of pixels included; 28 control cells with ~92% of pixels included), 7 minutes (18 UV cells with ~42% of pixels included; 28 control cells with ~90% of pixels included), and 9 minutes (11 UV cells with ~29% of pixels included; 28 control cells with ~88% of pixels included). The control cells are stable throughout the experiment with D=0.0053 ($\mu m^2\ s^{-1}$) (Supplementary Figure 5). UV irradiated cells start out at a high D = 0.0086 ($\mu m^2\ s^{-1}$) likely due to some UV exposure before the completion of the first measurement. The speed of molecular motion increases for the first five minutes to D = 0.017 ($\mu m^2\ s^{-1}$). Next, diffusion decreases for about two minutes to D = 0.015 ($\mu m^2\ s^{-1}$), stabilizes for a minute before sharply increasing to D = 0.020 ($\mu m^2\ s^{-1}$) at nine minutes.

Supplementary Note 5: Characterization of the Cellular Paroxysm

In general, the cellular paroxysm is characterized as a near-instantaneous, large scale burst of motion (transient increase in m_f across the whole cell) that is asynchronous from cell to cell. Individually, there can be variations in timing, localization, and synchronicity during the cellular paroxysm. To better characterize these variations in the paroxysm, we have performed a subsampling analysis to increase the temporal resolution of our dynamics measurements,

effectively decreasing the acquisition time from ~7 seconds to 0.7 seconds (details below). While this analysis doesn't improve the temporal resolution as much as the single frame analysis used in Figure 5bcd and Supplementary Movie 3, it allow us to output m_f maps, which the single frame analysis cannot, and identify features that are not clearly identified in the timing of cellular paroxysm maps (Figure 5d). Through this analysis, we have identified some interesting variations from the typical cellular paroxysm. Most cellular paroxysms occur across the entire cell, but occasionally we have observed partial paroxysms that only occur in one region of the cell (Supplementary Figure 6). In generally, the paroxysm initiates across the entire cell instantaneously, but we have observed a few cells that exhibit a wavelike initiation, where the paroxysm starts in one region of the cell and spreads to the rest of the cell over time (Supplementary Figure 7). Typically, paroxysms are asynchronous from cell to cell across the field of view, but sometimes adjacent cells synchronize and the paroxysms initiate simultaneously (Supplementary Figure 8). While the three previous features are relatively rare, cyclical paroxysms, where a smaller secondary burst of motion occurs after the initial paroxysm, appear regularly (Supplementary Figure 9). These secondary paroxysms can appear milliseconds to tens of seconds after the initial paroxysm. This analysis has shown that a significant amount of individual variation can occur within the cellular paroxysm phenomenon.

Subsampling Analysis Method

To increase the temporal resolution in post-processing, a single dynamics data cube is split along the time axis into ten separate data cubes reducing the acquisition time from 7.035 to 0.7035 seconds. Each of these reduced data cubes are analyzed using the standard algorithm to produce

m_f maps. This reduction in acquisition time increases noise in the m_f maps. A 2-D median spatial filter using a 3-by-3 super-pixel is applied to the maps to reduce this spatial noise.

Supplementary Note 6: UV Irradiation and Markers of Cell Death

Reduction in mitochondrial membrane potential, activation of caspase 3/7, and alterations in cell morphology are common features in the process of cell death. These features were tested under various lengths of UV treatment [control (*n* = 23), 1 minute (*n* = 20), 3 minutes (*n* = 21), 6 minutes (*n* = 16), and 20 minutes (*n* = 4)] and measured at 2.5 and 20 hours after UV irradiation. We observed a dosage dependent decrease in mitochondrial membrane potential when measured 2.5 hours after irradiation (Supplementary Figure 10). Caspase 3/7 activation was not observed for any UV dosage at with 2.5 or 20 hours after UV irradiation (Supplementary Figure 11). Note that while some signal is observed in these images, based on the localization and extremely low signal, the fluorescence seen in these images is likely to be autofluorescence and leakage from MitoTracker (Orange CMTMRos), not caspase 3/7 activation. Interestingly, morphological changes consistent with apoptosis were observed for low dosages of UV (1 and 3 minutes), but higher dosages of UV didn't show any change in morphology up to 20 hours later (Supplementary Figure 12). None of these features show any correlation with the cellular paroxysm.

Supplementary Note 7: UV Irradiation Length and Recovery

Continuous UV irradiation halts intracellular dynamics within 10-20 minutes, but it was unclear if the cell could recover from this damage and if that recovery would depend on the length of UV irradiation. To test this, HeLa cells were irradiated with UV for varying lengths of time [control (*n* = 35), 30 seconds (*n* = 39), 1 minute (*n* = 16), 3 minutes (*n* = 18), 6 minutes (*n* = 20), and 20

minutes (n = 11)], and remeasured at 3 and 20 hours after UV irradiation. Cells that underwent 20 minutes of UV irradiation experienced cellular paroxysms, while the rest of the dosages did not. The response of cells undergoing 20 and 6 minutes of irradiation were similar, intracellular dynamics stopped, never recovered, and there were no changes in cellular morphology (Supplementary Figure 13). The medium dosages of UV (1 and 3 minutes) also halted intracellular dynamics, but interestingly, some cells were able to restart their dynamics at 20 hours, while others were not (Supplementary Figure 14). Additionally, these cells displayed morphological changes consistent with apoptosis: cell shrinkage, fragmentation, and detachment from the glass substrate. The 30 second dosage showed a slightly decrease in dynamics, but was able to completely recover (Supplementary Figure 15). Overall, the length of UV irradiation affects the cellular response/recovery, and this recovery seems to be independent of the cellular paroxysm.

Supplementary Note 8: Dual-PWS Signal to Noise

Signal to Noise ratio (SNR) is an important consideration when acquiring dual-PWS images. Motion that produces a signal near or below the noise limit cannot be quantified. It should be noted the measurements of diffusion are particularly sensitive and will become inaccurate with low SNR. In general, SNR will be affected by the same parameters as any microscopy technique, but there are some special considers that should be considered.

Exposure time: SNR can be directly increased by increasing exposure time, but increased exposure time will reduce sensitively to faster processes (see Supplementary Note 2).

LED Intensity: SNR can be directly increased by increasing the input LED intensity. Theoretically, this will not affect the measurements in any other way, but high intensities of light can cause biological changes in cells (especially in the UV range). One potential solution is illumination side spectral filtering, which can reduce light intensity impacting the cells.

Camera Sensitivity and Noise: Improved camera sensitivity and reduced camera noise will improve SNR without any negative effects.

Cell Motion: The temporal interference signal originates from intracellular macromolecular dynamics. When comparing different cells lines or perturbations, increases or decreases in macromolecular dynamics will directly affect the SNR. As this is the parameter we are trying to measure, it cannot be tweaked to improve SNR, but it should be considered when planning and interpreting dual-PWS experiments.

Supplementary Note 9: FDTD Simulation

To further validate the experimental and theoretical basis of Σ_t, we used the Finite Difference Time Domain (FDTD) technique to simulate the microscope setup and experimental geometry indicated in section "Nanosphere Phantom Validation". This geometry consisted of a volume of randomly dispersed beads (RI = 1.60) in a homogenous media (RI = 1.46) mounted upon a sapphire slide (RI = 1.77), matching that of experimental setup (illumination and collection NA of 0.55 and 1.49 respectively). The initial time point started with a random initial position of beads. For every time point, each bead moved a randomly selected direction and distance based on a distribution that yielded an average diffusion coefficient appropriate for each sphere size and prohibited collisions

with other beads. Each time point was spaced 32ms apart and 42 time points were simulated in total for each bead size (100, 500, 37.5 and 25nm radii), for one volume fraction of 0.1%. The resulting time-varying geometries were used in Finite Difference Time Domain (FDTD) simulations to obtain spectral microscopic images. The FDTD simulations were performed using "*Angora*"[4]. FDTD time-marches electric-field and magnetic-field vector components mapped onto a spatial grid, thereby closely emulating electromagnetic wave propagation and scattering[5]. The backscattered waves were collected at the boundary of the spatial grid and transferred numerically to the image plane at the far zone. In these simulations the particles were placed in a 3-D cubic-cell FDTD space lattice of uniform cell size of 10 nm, terminated by a 5-cell-thick convolutional perfectly matched layer (CPML) absorbing boundary. Diffraction-limited microscopic images were constructed for desired wavelengths and Σ_t was calculated according to typical analysis method (Supplementary Figure 16).

Supplementary Figure 1

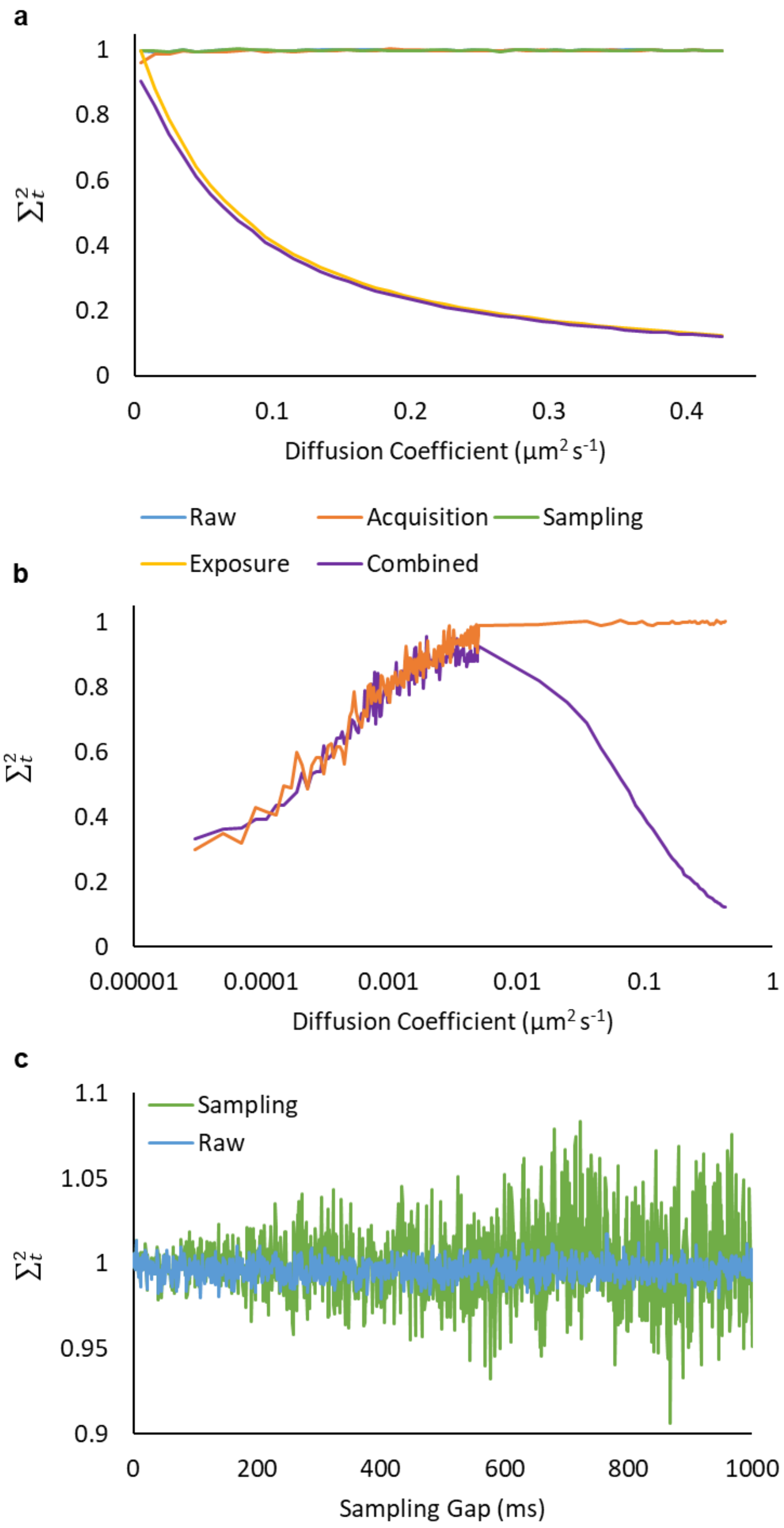
a
1
0.8
0.6
0.4
0.2
0
Σ_t^2
0
0.1
0.2
0.3
0.4
Diffusion Coefficient (μm^2 s^{-1})
Raw
Acquisition
Sampling
Exposure
Combined
b
1
0.8
0.6
0.4
0.2
0
Σ_t^2
0.00001
0.0001
0.001
0.01
0.1
1
Diffusion Coefficient (μm^2 s^{-1})
c
1.1
1.05
1
0.95
0.9
Σ_t^2
Sampling
Raw
0
200
400
600
800
1000
Sampling Gap (ms)

Supplementary Figure 1: Effect of Experimental Timing on Σ_{t}^{2}. (a) The effect of exposure time, acquisition time, and sampling on Σ_{t}^{2} for parameters used in the system validation phantom measurements: 32 ms exposure, 32 ms sampling, and 6.4 s acquisition time. (b) Expanding the range to extremely slow diffusion coefficients to see the effect of acquisition time on Σ_{t}^{2}. (c) Testing a large range of sampling rates to see the effect of sampling on Σ_{t}^{2}.

Supplementary Figure 2

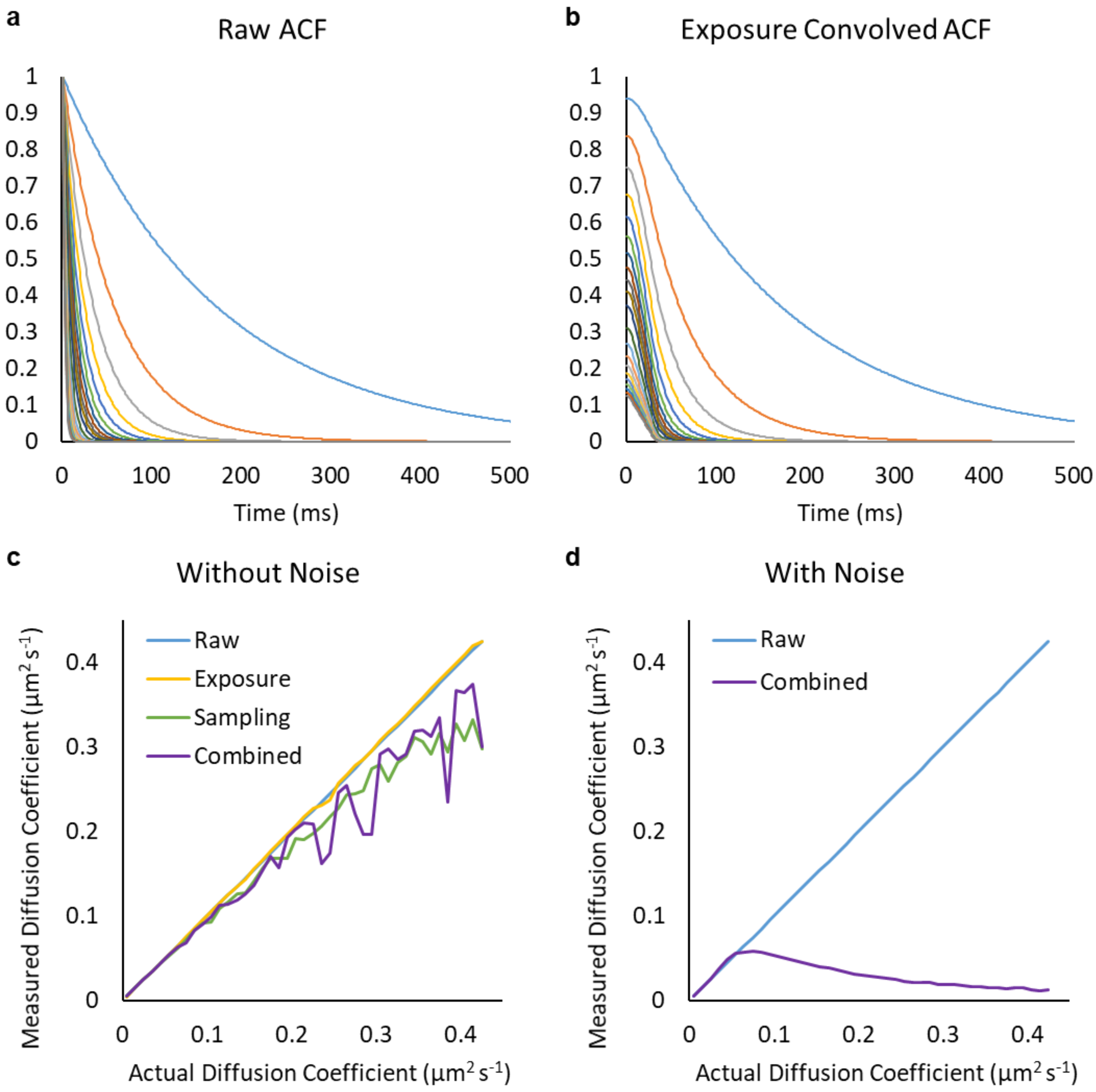

Supplementary Figure 2: Effect of Experimental Timing on *D*. (a) Exponential decays as theoretical autocorrelation functions (ACFs) without noise for *D*: 0.005 $\mu m^2 s^{-1} < D < 0.45 \mu m^2 s^{-1}$ (b) Exponential decays as theoretical ACFs without noise after convolution with a triangular function to model the effects of exposure time for *D*: 0.005 $\mu m^2 s^{-1} < D < 0.45 \mu m^2 s^{-1}$. (c/d) The effect of exposure time and sampling on *D* without noise (c) and with noise (d) for parameters

used in the system validation phantom measurements: 32 ms exposure, 32 ms sampling, and 6.4 s acquisition time.

Supplementary Figure 3

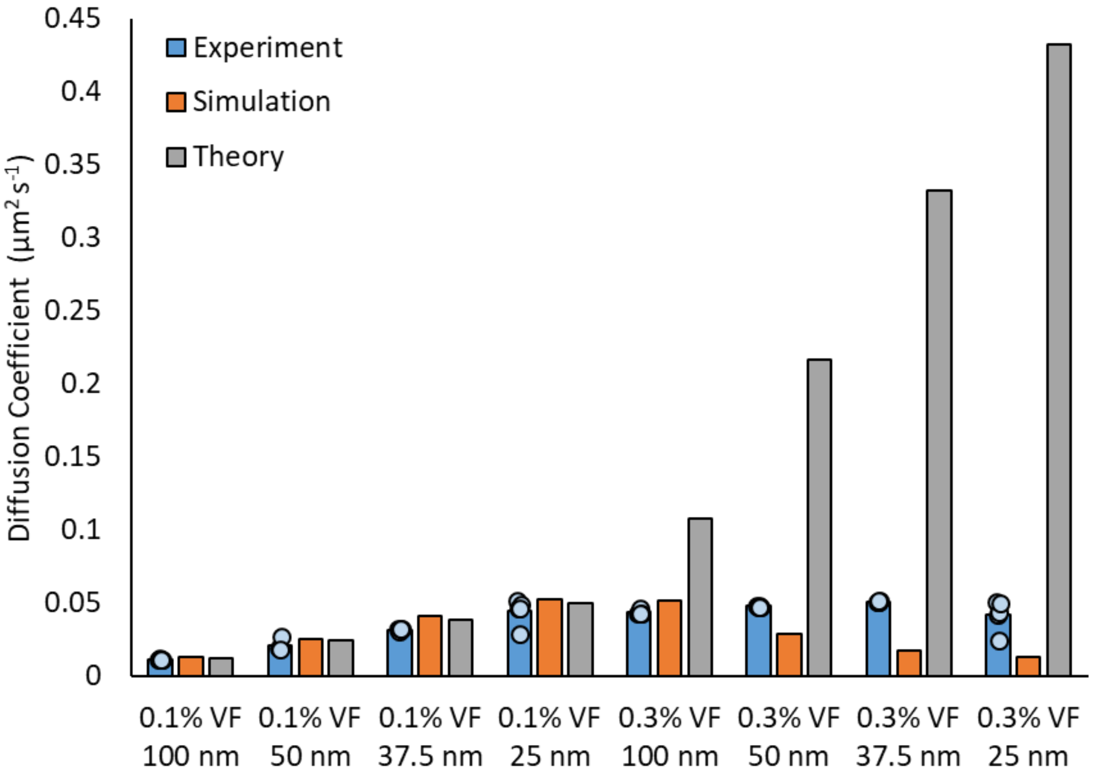

Supplementary Figure 3: Full Phantom Diffusion Comparison. Bar graph with individual data points comparing experimentally measured *D*, theoretical values calculated using the Stokes-Einstein equation, and simulated results taking exposure time, acquisition time, and sampling into account for both 0.1% VF (*Φ*) and 0.3% VF (*Φ*) sphere phantoms with sizes 25nm, 37.5nm, 50nm, and 100nm.

Supplementary Figure 4

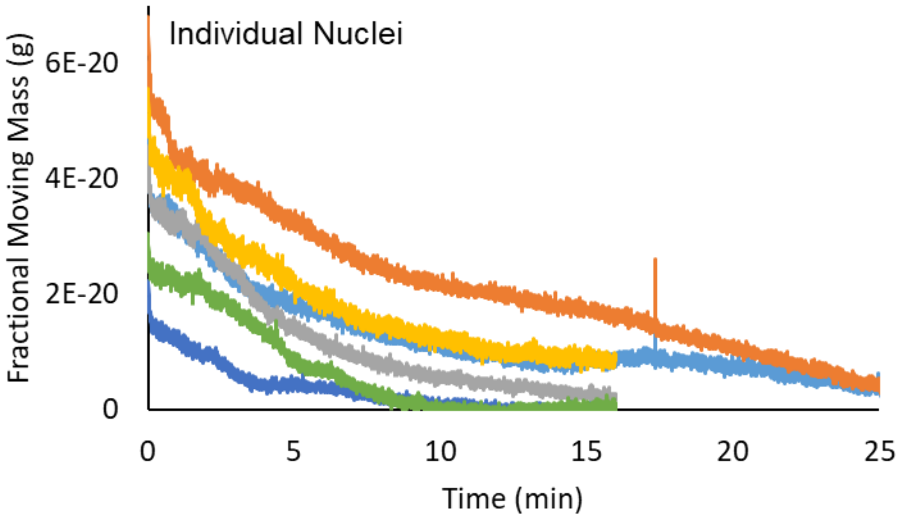

Supplementary Figure 4: High Temporal Resolution Ultraviolet Irradiation Quantification. Line graphs showing individual cell response to UV irradiation within the nucleus from the high temporal resolution system.

Supplementary Figure 5

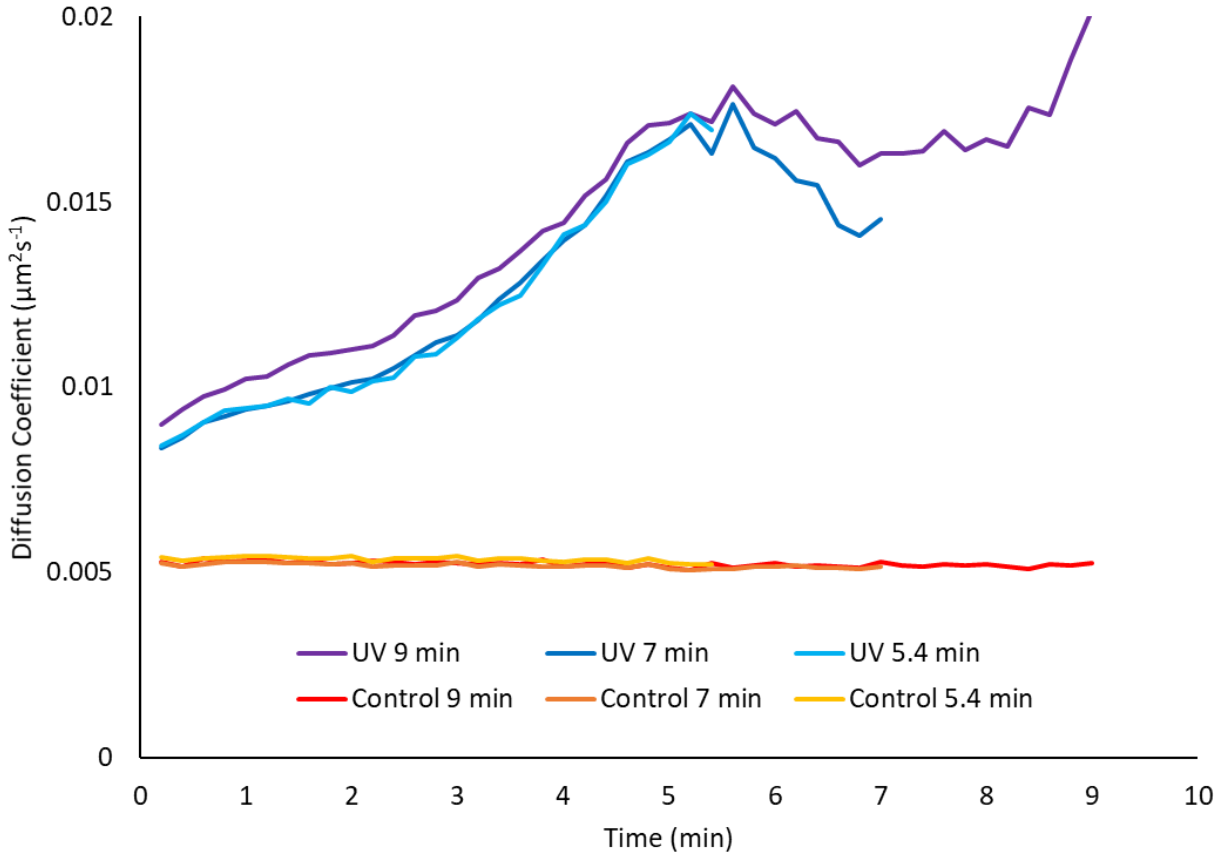

Supplementary Figure 5: Ultraviolet Irradiation Diffusion Quantification. Line graph showing the averaged *D* in response to UV and non-UV irradiation within the nucleus. For this analysis there is a tradeoff between the length of the experiment analyzed, the number of pixels per cell that meet the noise threshold, and the number of cells that meet the pixel threshold. Therefore, *D* was calculated for three different experiment lengths.

Supplementary Figure 6

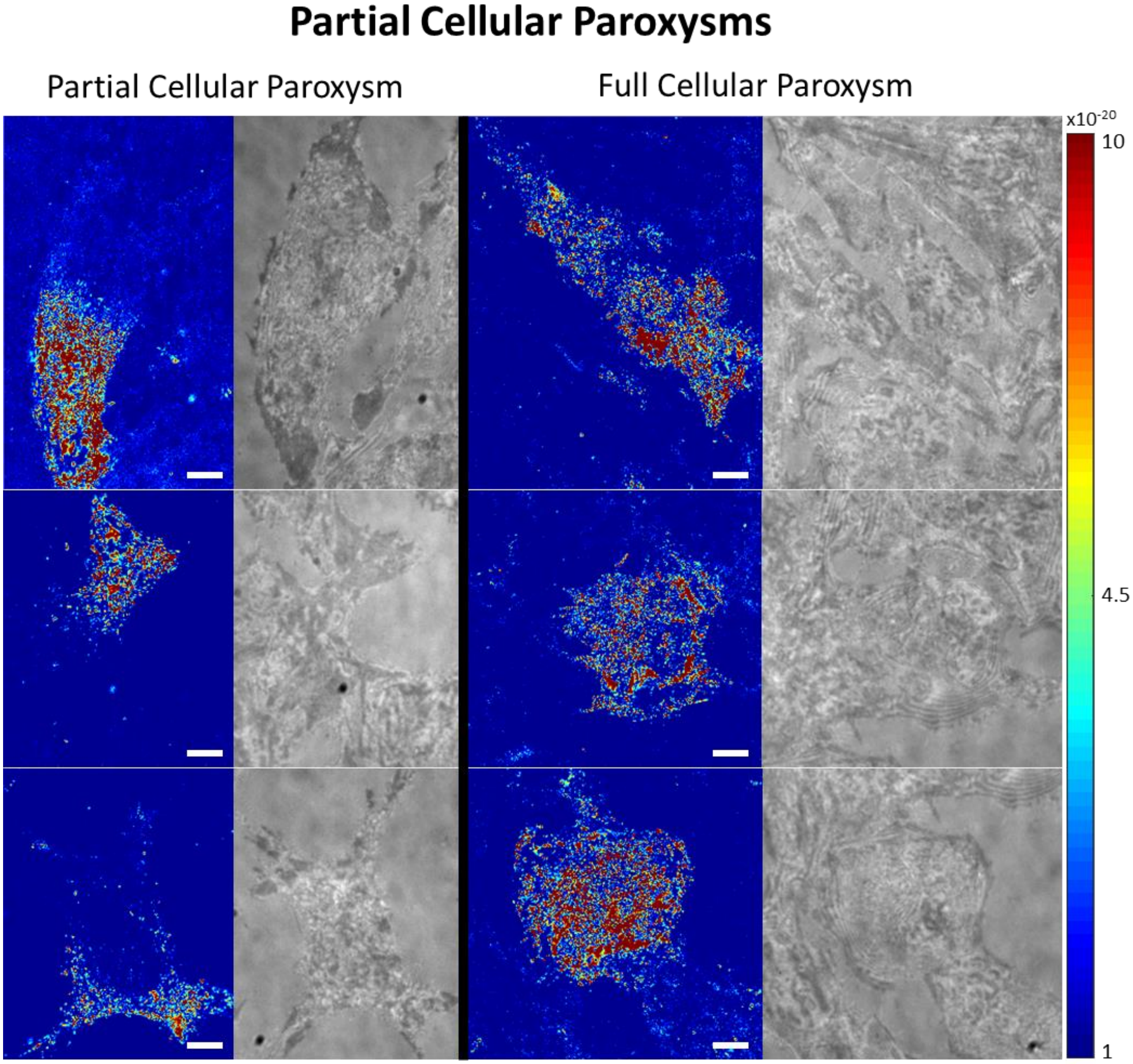

Supplementary Figure 6: Partial Cellular Paroxyms. Partial paroxysms are one of the features identified during the characterization of cellular paroxysms. Generally, paroxysms occur within the entirety of the cell, but occasionally, they will only effect a small section of the cell. Scale bar is 8 µm.

Supplementary Figure 7

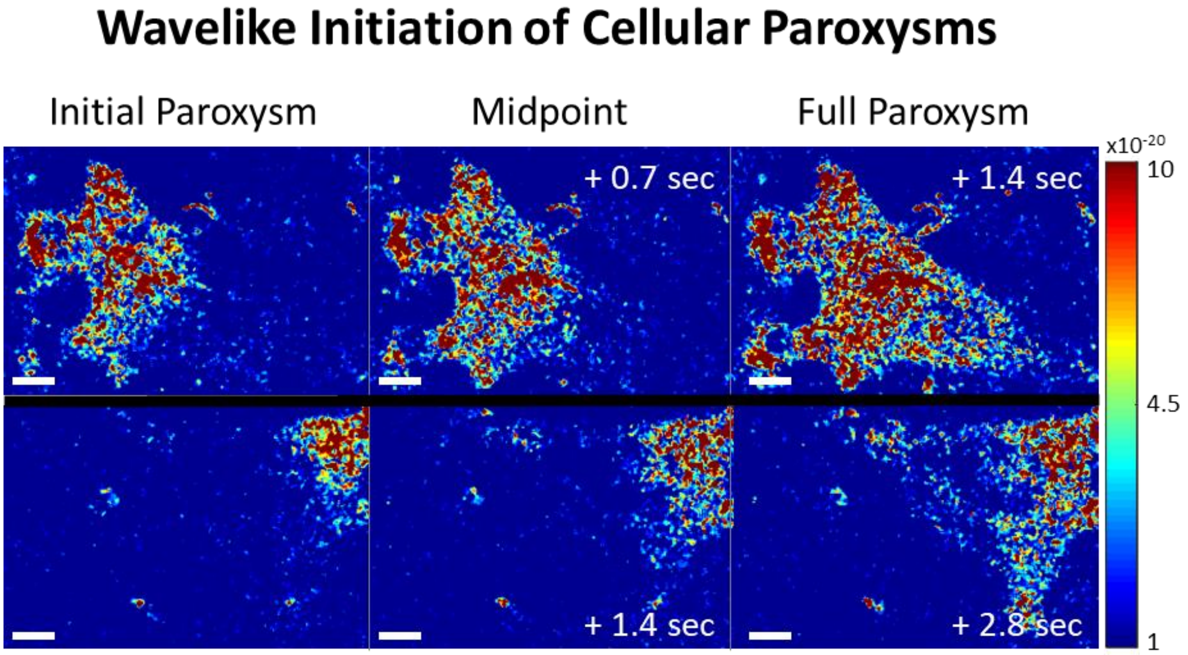

Supplementary Figure 7: Wavelike Initiation of Cellular Paroxyms. Wavelike initiation of the cellular paroxym is one of the features identified during the characterization of cellular paroxysms. Generally, paroxysms initiate across the entirety of the cell instantaneously, but occasionally, they will initiate in one part of the cell and spread out in a wavelike manner across the entire cell. Scale bar is 8 µm.

Supplementary Figure 8

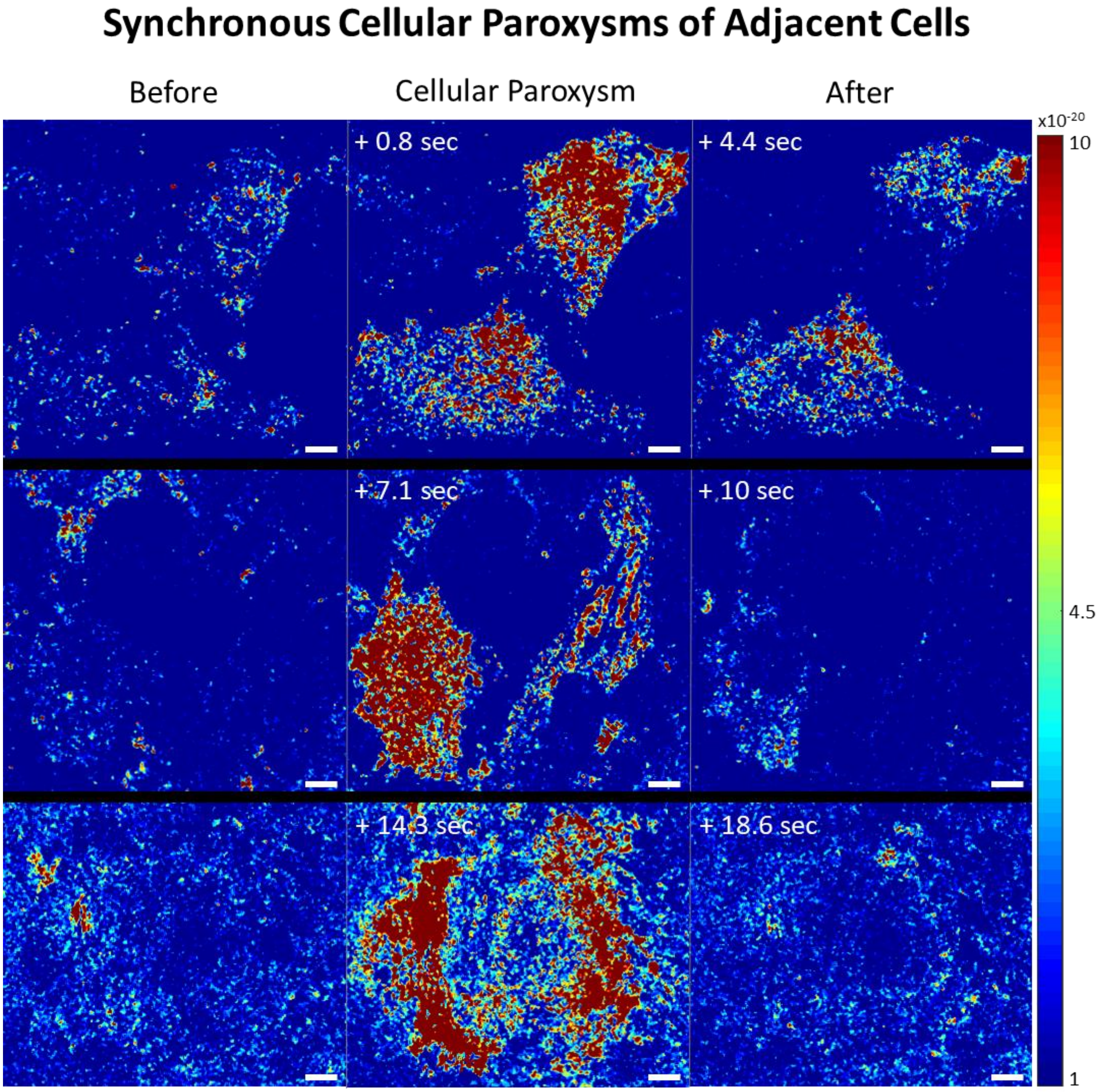

Supplementary Figure 8: Synchronous Cellular Paroxyms. Synchronous cellular paroxyms are one of the features identified during the characterization of cellular paroxysm. Generally, paroxysms initiate asynchronously from cell to cell, but occasionally, adjacent cells (generally in contact with each other) will undergo cellular paroxysm simultaneously. Scale bar is 8 µm.

Supplementary Figure 9

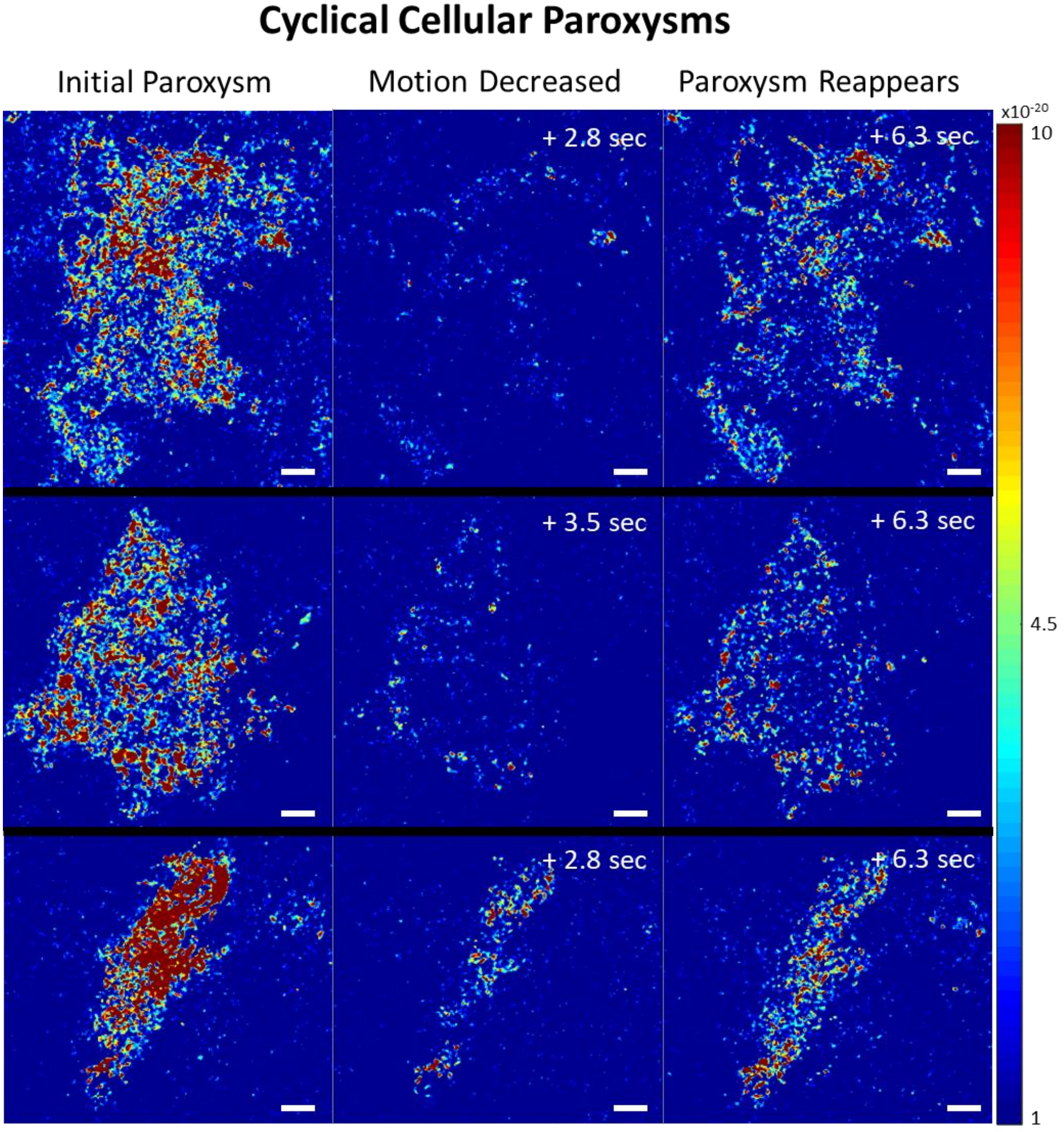

Supplementary Figure 9: Cyclical Cellular Paroxyms. Cyclical cellular paroxyms are one of the features identified during the characterization of cellular paroxysm. Sometimes, after the initial

cellular paroxysm, a secondary smaller paroxysm will occur milliseconds to tens of seconds after the initial paroxysm has subdued. Scale bar is 8 μm.

Supplementary Figure 10

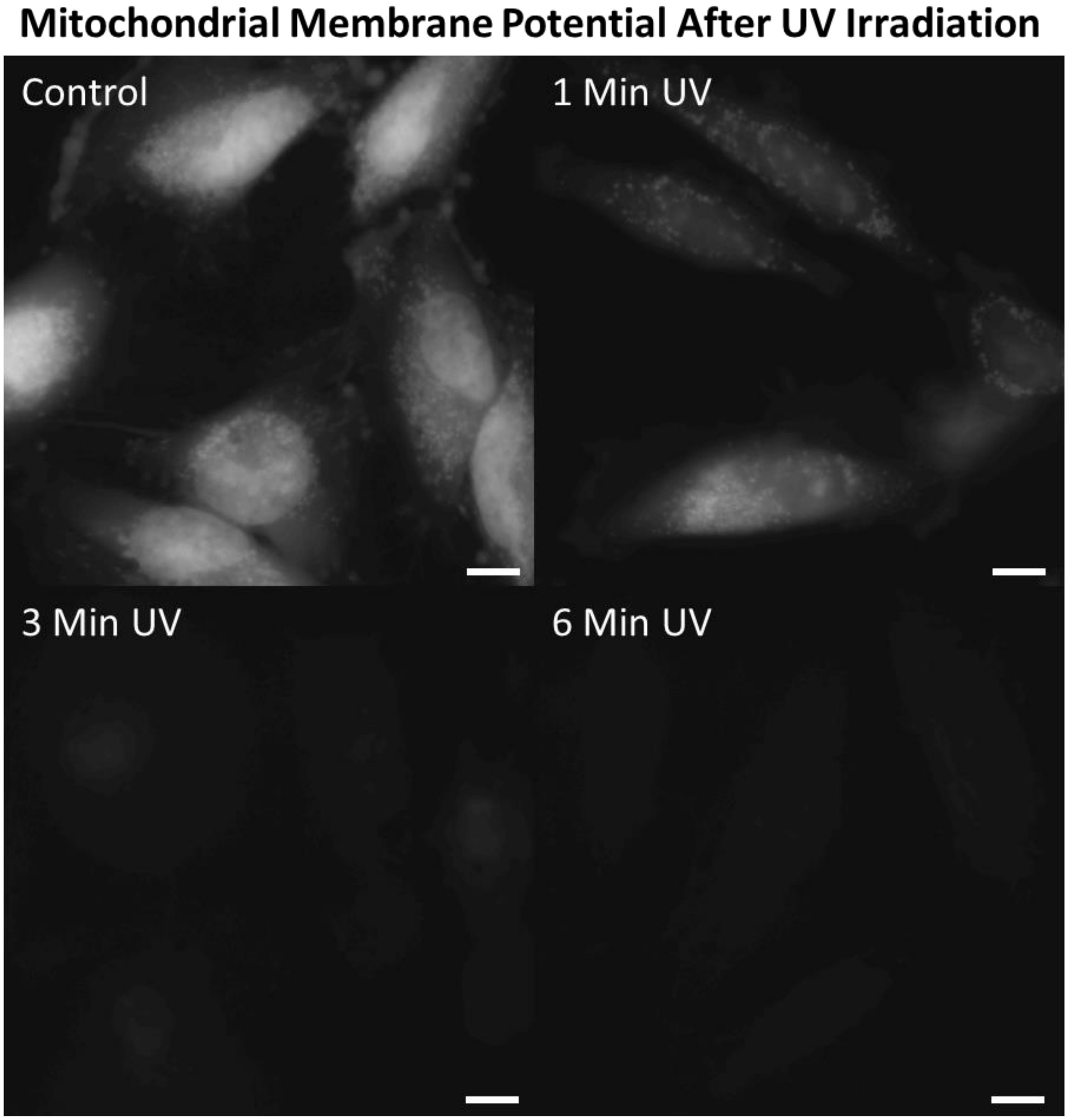

Supplementary Figure 10: Mitochondrial Membrane Potential After UV Irradiation. MitoTracker Orange CMTMRos fluorescence images from HeLa cells 2.5 hours after various lengths of UV

irradiation (no irradiation, 1 minute, 3 minutes, and 6 mintues). UV irradiation reduces mitochondrial membrane potential. Scale bar is 13 µm.

Supplementary Figure 11

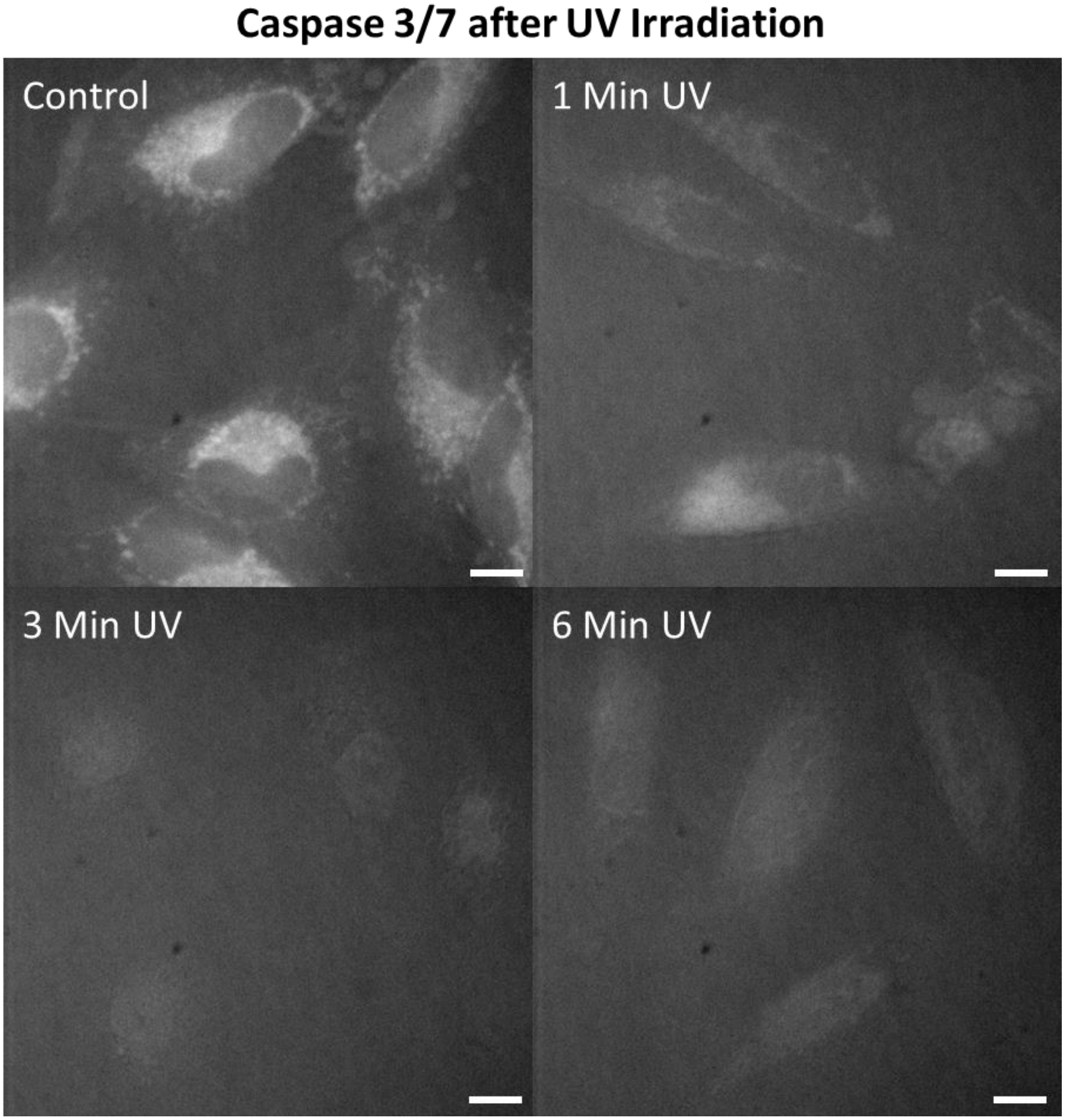

Supplementary Figure 11: Caspase 3/7 Activation After UV Irradiation. CellEvent Caspase-3/7 fluorescence images from HeLa cells 2.5 hours after various lengths of UV irradiation (no

irradiation, 1 minute, 3 minutes, and 6 minutes). UV irradiated cells did not exhibit caspase 3/7 activation 2.5 hours after UV irradiation. While some signal is observed in these images, based on the localization and extremely low signal, the fluorescence seen in these images is likely to be autofluorescence and leakage from MitoTracker, not caspase 3/7 activation. Scale bar is 13 μm.

Supplementary Figure 12

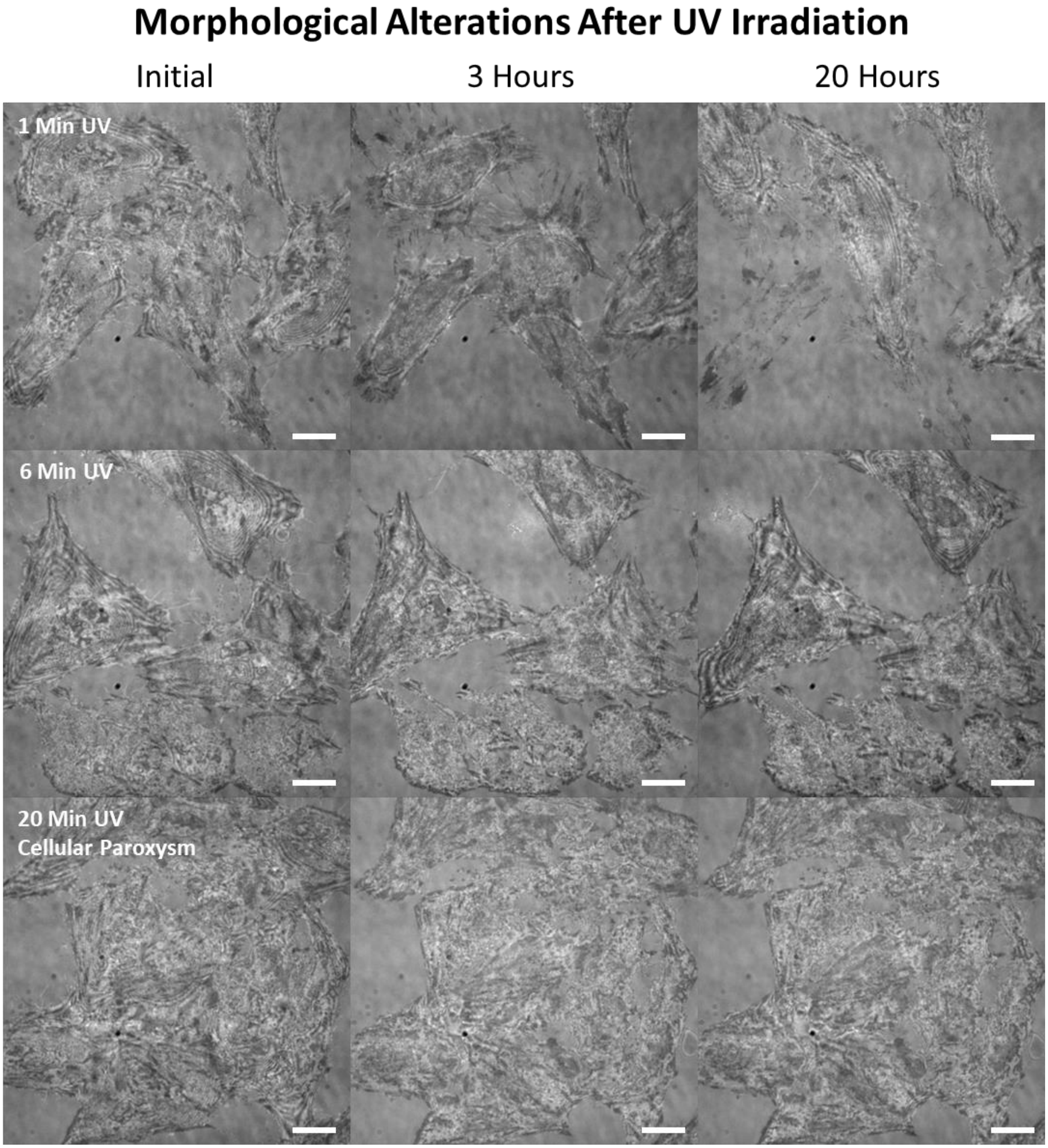

Supplementary Figure 12: Morphological Changes Due To UV Irradiation. Representative reflectance microscopy images showing morphological changes from HeLa cells that were irradiated with UV for 1 minute (top), 6 minutes (middle), and 20 minutes (bottom) at time points

before irradiation (left), 3 hours after irradiation (middle), and 20 hours after irradiation (right). Low dosage UV irradiation induces morphological changes consistent with apoptosis, while cells undergoing higher UV dosages don't show any morphological changes up to 20 hours later. Scale bar is 19 µm.

Supplementary Figure 13

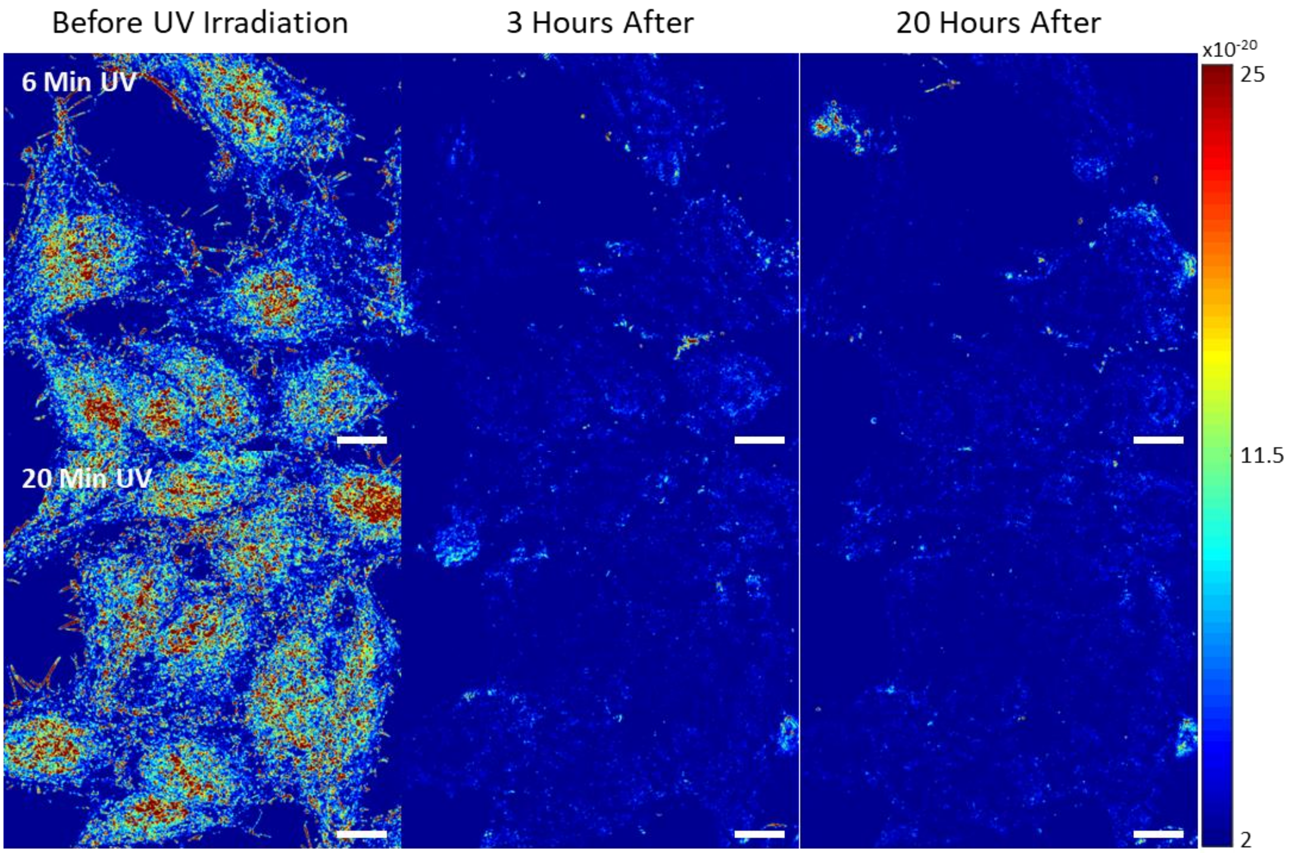

Supplementary Figure 13: Long Dosage UV Irradiation and Recovery. Representative m_f maps of HeLa cells that were irradiated with UV for 6 minutes (top) and 20 minutes (bottom) at time points before irradiation (left), 3 hours after irradiation (middle), and 20 hours after irradiation (right). After long dosages of UV cells are unable to recover any macromolecular motion up to 20 hours later. Scale bar is 19 μm.

Supplementary Figure 14

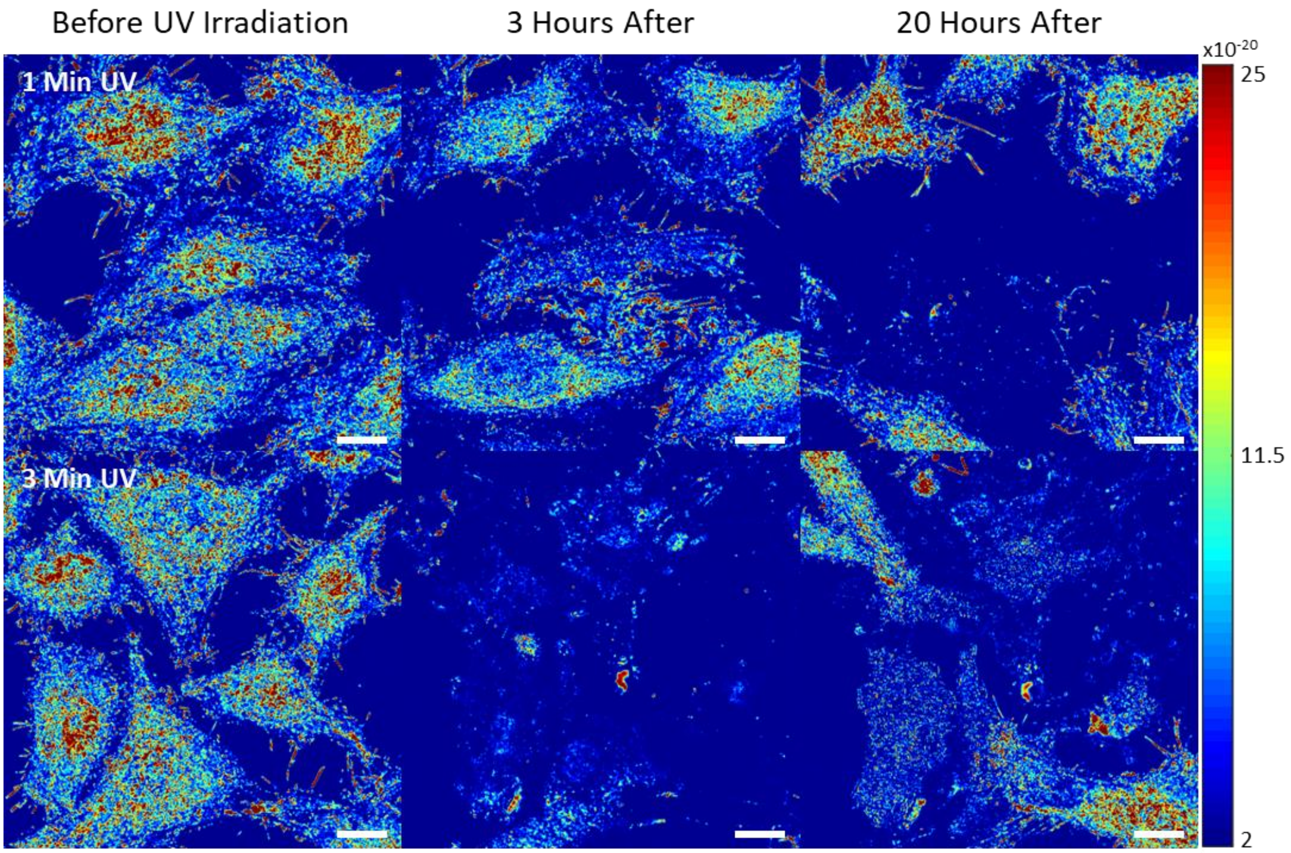

Supplementary Figure 14: Medium Dosage UV Irradiation and Recovery. Representative m_f maps of HeLa cells that were irradiated with UV for 1 minute (top) and 3 minutes (bottom) at time points before irradiation (left), 3 hours after irradiation (middle), and 20 hours after irradiation (right). After medium dosages of UV some cells are able to partially recover macromolecular motion. Scale bar is 19 µm.

Supplementary Figure 15

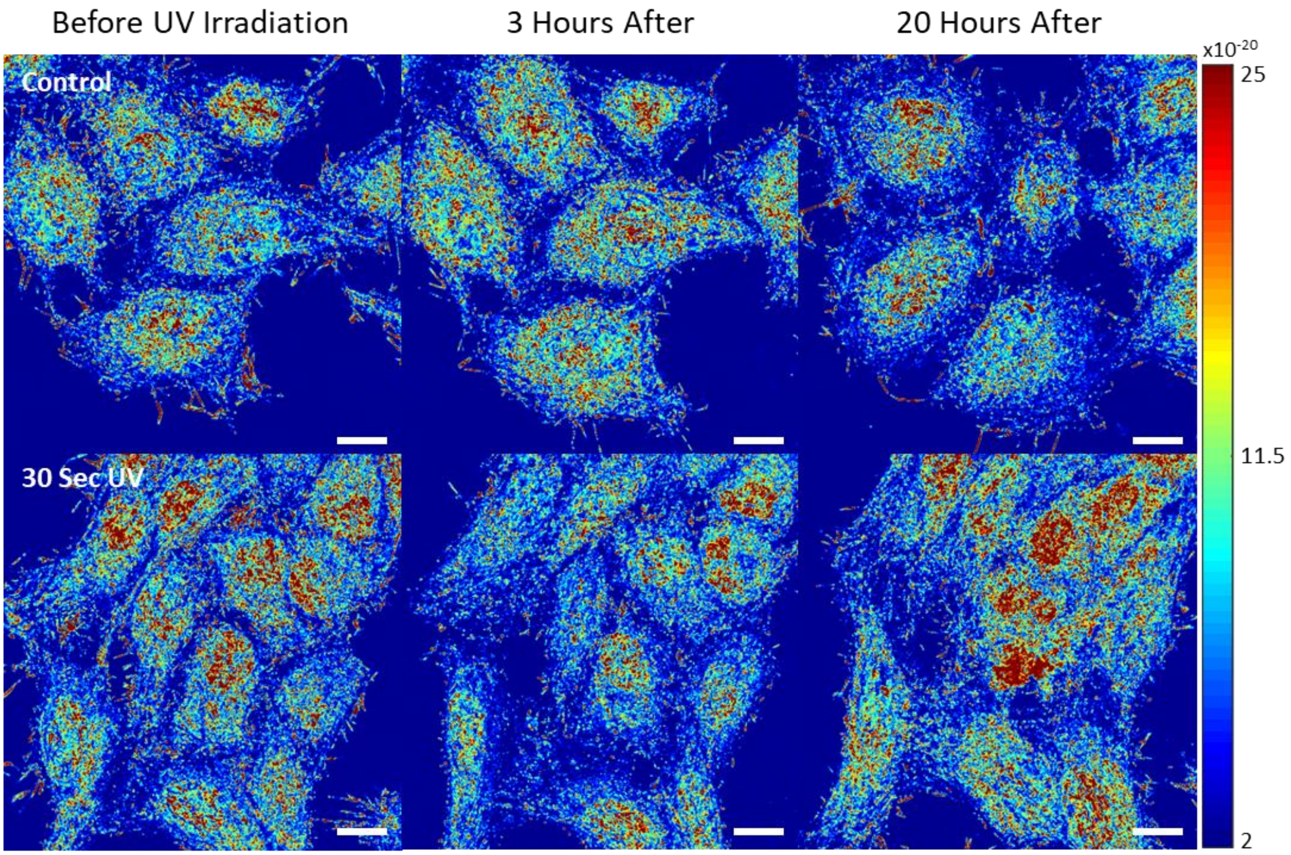

Supplementary Figure 15: Control and Low Dosage UV Irradiation and Recovery. Representative m_f maps of HeLa cells didn't undergo irradiation (top) and cells that were irradiated with UV for 30 seconds (bottom) at time points before irradiation (left), 3 hours after irradiation (middle), and 20 hours after irradiation (right). Control cells and low dosage UV irradiation do not cause significant changes in macromolecular motion. Scale bar is 19 µm.

Supplementary Figure 16

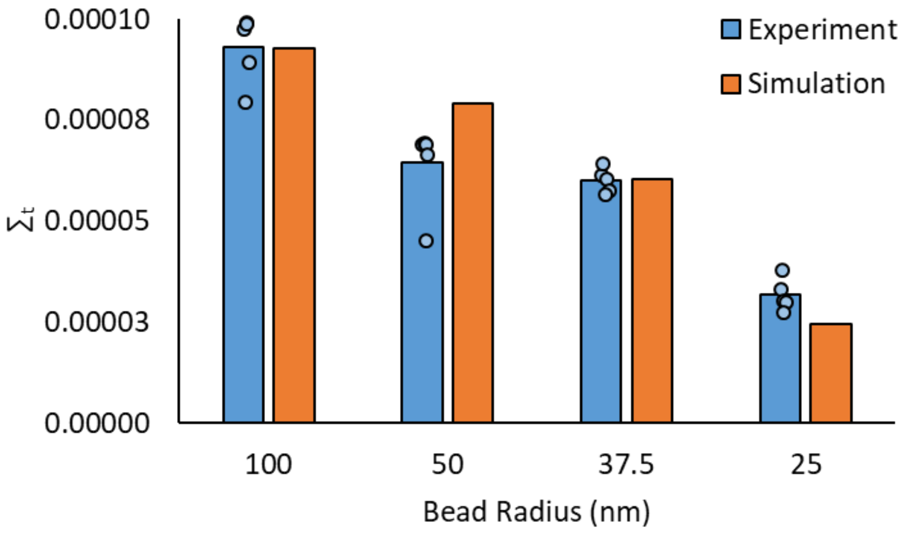

Supplementary Figure 16: FDTD Simulations of Nanosphere Phantoms. Bar graph with individual data points comparing Σ_t calculated from experimental nanosphere phantoms and Finite Difference Time Domain (FDTD) simulations of diffusing spheres of various sizes. Experimental and simulation data match well with $R^2 = 0.91$.

Supplementary Figure 17

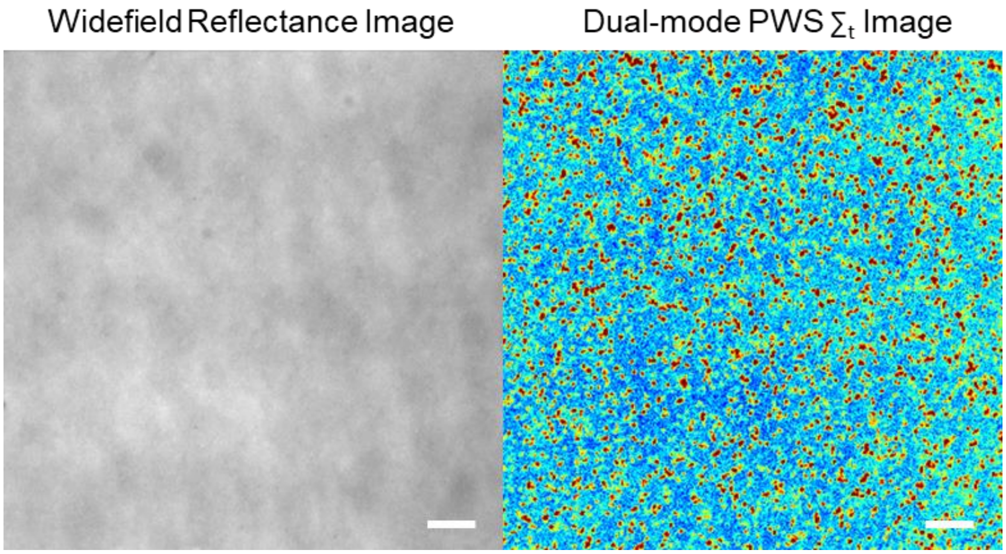

Supplementary Figure 17: Nanosphere Phantoms Images. Widefield reflectance image (left) and Σ_t map (right) of 100nm 0.1% volume fraction polystyrene nanosphere phantom. Due to diffraction limited resolution of traditional microscopy the nanospheres cannot be visualized in the widefield reflectance image. Scale bar is 8 uM.

Supplementary Figure 18

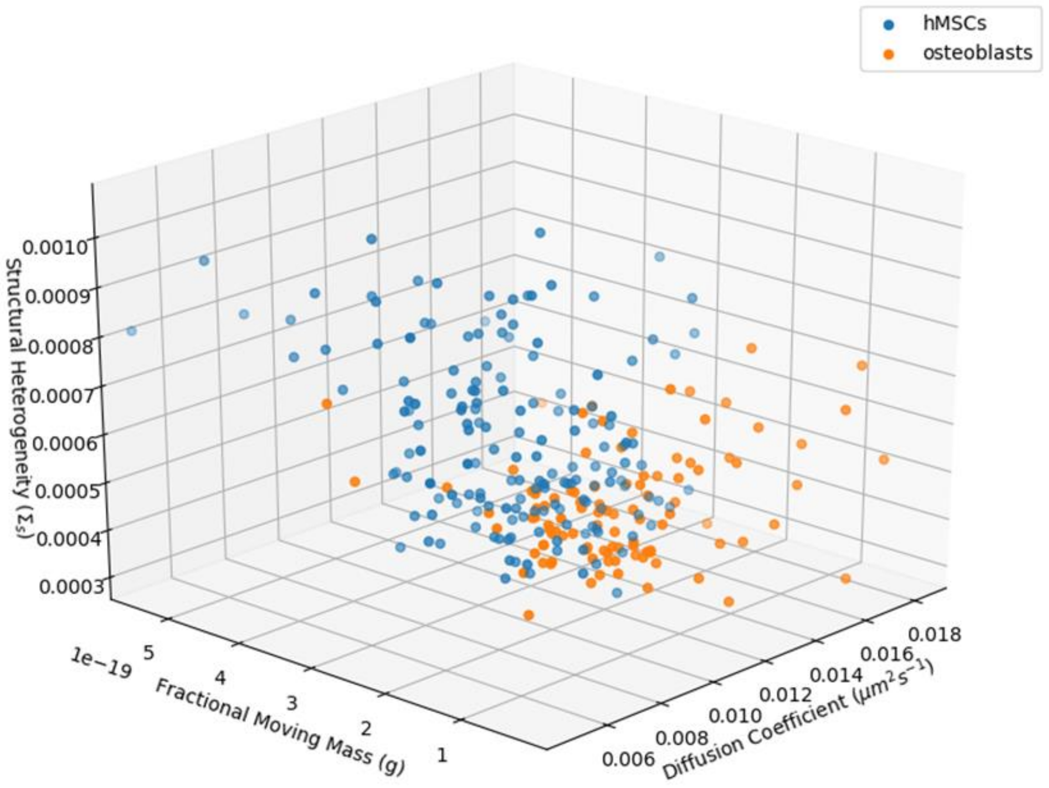

Supplementary Figure 18: Stem Cell Structure-Dynamics Distribution. 3D scatter plot showing the overall distribution of the hMSC and osteoblast populations within this structure-dynamics space. While the populations are heterogeneous with some overlap, the chromatin folding and nuclear dynamics states are significantly different between the populations. (*n* = 166 hMSC and *n* = 102 osteoblasts)

Supplementary References

1 Cherkezyan, L. *et al.* Interferometric Spectroscopy of Scattered Light Can Quantify the Statistics of Subdiffractional Refractive-Index Fluctuations. *Physical Review Letters* **111**, 033903 (2013).

2 Hu, C. & Popescu, G. Physical significance of backscattering phase measurements. *Opt. Lett.* **42**, 4643-4646, doi:10.1364/OL.42.004643 (2017).

3 Berne, B. J. & Pecora, R. *Dynamic light scattering : with applications to chemistry, biology, and physics*. 58 (Dover Publications, 1976).

4 Çapoğlu, I. R., Taflove, A. & Backman, V. Angora: A Free Software Package for Finite-Difference Time-Domain Electromagnetic Simulation. *IEEE Antennas and Propagation Magazine* **55**, 80-93, doi:10.1109/MAP.2013.6645144 (2013).

5 Taflove, A. & Hagness, S. C. *Computational Electrodynamics: The Finite-Difference Time-Domain Method*. (Artech House, 2000).
