# Supplementary material for: Multimodal interference-based imaging of nanoscale structure and macromolecular motion uncovers UV induced cellular paroxysm
Source: Nat Commun. 2019 Apr 10;10:1652. doi: 10.1038/s41467-019-09717-6 (PMC6458150; doi:10.1038/s41467-019-09717-6)
Supplement: Supplementary file 3 — Description of Additional Supplementary Files [file 41467_2019_9717_MOESM3_ESM.pdf]

Description of Additional Supplementary Files

File Name: Supplementary Movie 1
Description: High Temporal Resolution System Video. High temporal resolution mf videos for cells undergoing UV irradiation.

File Name: Supplementary Movie 2
Description: Ultraviolet Irradiation Movie. Representative mf (top) and (bottom) sequences for cells undergoing UV (right) and non-UV (left) irradiation. Cellular paroxysms can be observed in the UV irradiated mf sequence after 12 minutes of irradiation.

File Name: Supplementary Movie 3
Description: Cellular Paroxysm Timing Localization. Movie showing the timing of cellular paroxysms for each pixel within a representative cell. The spatial distribution of this timing shows that the initial motion occurs simultaneously within a single 35ms frame on opposite ends of the cell.
