# Supplementary material for: Multimodal interference-based imaging of nanoscale structure and macromolecular motion uncovers UV induced cellular paroxysm
Source: Nat Commun. 2019 Apr 10;10:1652. doi: 10.1038/s41467-019-09717-6 (PMC6458150; doi:10.1038/s41467-019-09717-6)
Supplement: Supplementary file 7 — Reporting Summary Checklist [file 41467_2019_9717_MOESM7_ESM.pdf]

nature research

Corresponding author(s): Vadim Backman

Last updated by author(s): Mar 12, 2019

# Reporting Summary

Nature Research wishes to improve the reproducibility of the work that we publish. This form provides structure for consistency and transparency in reporting. For further information on Nature Research policies, see Authors & Referees and the Editorial Policy Checklist.

## Statistics

For all statistical analyses, confirm that the following items are present in the figure legend, table legend, main text, or Methods section.

| n/a | Confirmed | |
|---|---|---|
| ☐ | ☒ | The exact sample size (*n*) for each experimental group/condition, given as a discrete number and unit of measurement |
| ☐ | ☒ | A statement on whether measurements were taken from distinct samples or whether the same sample was measured repeatedly |
| ☐ | ☒ | The statistical test(s) used AND whether they are one- or two-sided<br>*Only common tests should be described solely by name; describe more complex techniques in the Methods section.* |
| ☒ | ☐ | A description of all covariates tested |
| ☒ | ☐ | A description of any assumptions or corrections, such as tests of normality and adjustment for multiple comparisons |
| ☐ | ☒ | A full description of the statistical parameters including central tendency (e.g. means) or other basic estimates (e.g. regression coefficient) AND variation (e.g. standard deviation) or associated estimates of uncertainty (e.g. confidence intervals) |
| ☐ | ☒ | For null hypothesis testing, the test statistic (e.g. *F*, *t*, *r*) with confidence intervals, effect sizes, degrees of freedom and *P* value noted<br>*Give P values as exact values whenever suitable.* |
| ☒ | ☐ | For Bayesian analysis, information on the choice of priors and Markov chain Monte Carlo settings |
| ☒ | ☐ | For hierarchical and complex designs, identification of the appropriate level for tests and full reporting of outcomes |
| ☐ | ☒ | Estimates of effect sizes (e.g. Cohen's *d*, Pearson's *r*), indicating how they were calculated |

*Our web collection on statistics for biologists contains articles on many of the points above.*

## Software and code

Policy information about availability of computer code

| | |
|---|---|
| Data collection | Dual-PWS data was acquired using custom code built in Matlab R2015b. Description of the data acquisition process is included in the methods section of the manuscript. Code can be provided upon request. |
| Data analysis | Biomakers were calculated using custom code built in Matlab R2015b. Description of the data analysis is included in the methods section of the manuscript. Code can be provided upon request. Additional statistical analysis was performed using Microsoft Excel 2016. |

For manuscripts utilizing custom algorithms or software that are central to the research but not yet described in published literature, software must be made available to editors/reviewers. We strongly encourage code deposition in a community repository (e.g. GitHub). See the Nature Research guidelines for submitting code & software for further information.

## Data

Policy information about availability of data

All manuscripts must include a data availability statement. This statement should provide the following information, where applicable:
- Accession codes, unique identifiers, or web links for publicly available datasets
- A list of figures that have associated raw data
- A description of any restrictions on data availability

All analyzed data and data statistics supporting the findings of this study are contained within the manuscript and its Supporting Information files. Raw data is available from the corresponding author upon reasonable request.

# Field-specific reporting

Please select the one below that is the best fit for your research. If you are not sure, read the appropriate sections before making your selection.

☒ Life sciences ☐ Behavioural & social sciences ☐ Ecological, evolutionary & environmental sciences

For a reference copy of the document with all sections, see nature.com/documents/nr-reporting-summary-flat.pdf

# Life sciences study design

All studies must disclose on these points even when the disclosure is negative.

| | |
|---|---|
| Sample size | Sample size was determined by calculating the number of cells needed so that the standard error of the mean was 10% or less for the measured biomarker at the initial stage of the experiment. |
| Data exclusions | The full time course UV Irradiation data was preformed in triplicate, a single representative replicate was presented in the manuscript as the experimental timing between the experiments was not exactly the same, so the data was not combined. The trends were the same between all replicates. |
| Replication | All experiments unless otherwise noted were performed at least 3 times and all replicates were successful. Fixation was only performed once as it was just used to demonstrate the absence of motion due to fixation, which is a widely accepted consequence of chemical fixation. |
| Randomization | Experimental and control dishes were selected at random at the beginning of each applicable experiment. |
| Blinding | Blinding was not possible during data acquisition for biological experiments as the procedures required actions like removing the UV filter, adding PFA, or other steps that made blinding not feasible. |

# Reporting for specific materials, systems and methods

We require information from authors about some types of materials, experimental systems and methods used in many studies. Here, indicate whether each material, system or method listed is relevant to your study. If you are not sure if a list item applies to your research, read the appropriate section before selecting a response.

## Materials & experimental systems

| n/a | Involved in the study | |
|---|---|---|
| ☐ | ☒ | Antibodies |
| ☐ | ☒ | Eukaryotic cell lines |
| ☒ | ☐ | Palaeontology |
| ☒ | ☐ | Animals and other organisms |
| ☒ | ☐ | Human research participants |
| ☒ | ☐ | Clinical data |

## Methods

| n/a | Involved in the study | |
|---|---|---|
| ☒ | ☐ | ChIP-seq |
| ☒ | ☐ | Flow cytometry |
| ☒ | ☐ | MRI-based neuroimaging |

## Antibodies

| | |
|---|---|
| Antibodies used | Cell Signaling Technology BrdU Mouse monoclonal antibody (Bu20a) Product Number: 5292; Invitrogen Alexa Fluor 532 Goat Anti-mouse IgG lot# 1857666 ref #:A1002 |
| Validation | Cell Signaling Technology provides a certificate of analysis certifying the applicability of the BrdU (Bu20a) for Immunofluorescence; Alexa Fluor 532 Goat Anti-mouse antibody was test against human IgG and human serum prior to conjugation and was certified for use in immunofluorescece. |

## Eukaryotic cell lines

Policy information about cell lines

| | |
|---|---|
| Cell line source(s) | All cell lines were purchased from ATCC. |
| Authentication | None |
| Mycoplasma contamination | HeLa cells were negative for Mycoplasma contamination. hMSC cells were not tested. |
| Commonly misidentified lines (See ICLAC register) | None |
